# Risk and Protective Factors Associated to Peer School Victimization

**DOI:** 10.3389/fpsyg.2017.00441

**Published:** 2017-03-22

**Authors:** Inmaculada Méndez, Cecilia Ruiz-Esteban, J. J. López-García

**Affiliations:** ^1^Department of Developmental Psychology and Education, University of MurciaMurcia, Spain; ^2^Department of Basic Psychology and Methodology, University of MurciaMurcia, Spain

**Keywords:** bullying, secondary education, adolescence, drug consumption, peers, family

## Abstract

The main objective of this study is to analyze the relationship between peer school victimization and some risk and protection factors and to compare the differences by role in victimization with those of non-involved bystanders. Our participants were 1,264 secondary students (*M* = 14.41, *SD* = 1.43) who participated voluntarily, although an informed consent was requested. A logistic regression model (LR) was used in order to identify the victim’s potential risks and protective factors related to non-involved bystanders. A multiple LR and a forward stepwise LR (Wald) were used. The results showed the variables related to the victim profile were: individual features (to be male, to be at the first cycle of compulsory Secondary Education and a few challenging behaviors), school environments (i.e., school adjustment), family environment (parental styles like authoritarianism) and social environment (i.e., friends who occasionally show a positive attitude toward drug consumption and easy access to drugs, access to drugs perceived as easy, rejection by peers or lack of social acceptance and social maladjustment). The results of the study will allow tackling prevention and intervention actions in schools, families, and social environment in order to improve coexistence at school and to assist the victimized students in the classroom.

## Introduction

Among the problems that arise at school ages, there may appear situations of harassment or bullying (Ortega-Ruiz, 2015), that is, an aggressive and intentional attack carried out repeatedly and overtime by a group or an individual against a victim who cannot easily fight back; or in other words, a power imbalance (Olweus, 1993, 2013). This problem makes no distinctions between geographic location, social status, public or private schools, etc. The report issued by Save the Children in Spain (Sastre, 2016) reveals that 9.3% of students have ever been bullying victims. Moreover, 5.4% of them admitted to have been bullied. There are three groups of key actors involved in bullying: aggressor, victim, provocative victim (Olweus, 2013). Literature also reinforces the key role of non-involved bystanders in bullying dynamics. Bystanders not involved in the action can take on different roles (Sullivan et al., 2005): accomplices, boosters, non-involved bystanders and defenders). Out of fear of clashing with the aggressor, some students become morally involved in false rules of silence (Ortega, 2000; Armas, 2007). Thus, the main objective of this study is to analyze the relationship between peer school victimization and some risk and protection factors and the differences by role in victimization comparing them with those of the non-involved bystanders in the action. There are different risks or protective factors, both personal and contextual or environmental, that accelerate victimization or make it more likely to happen.

With regards to social environment, examples of risk factors that can be associated with peers include at interpersonal level: peer group as pattern of submission and need of acceptance (Sullivan et al., 2005); modeling (Sánchez et al., 2007; Alfonso et al., 2009; Pérez-Fuentes and Gázquez, 2010; Delegación del Gobierno para el Plan Nacional sobre Drogas [DGPNSD], 2014), especially through the best friend (Espada et al., 2008); promoting access to drug use (Cerezo et al., 2013); the existence of drugs in the social environment which implies their accessibility, their visibility and their availability together with the attitude of society toward drugs (Delegación del Gobierno para el Plan Nacional sobre Drogas [DGPNSD], 2007).

Several studies have found that victimization is related to multiple variables related to individual features too. For instance, it has been observed that the possibility of becoming a victim depends on some variables. Some personal features are: to be younger than the aggressors and the average classmate (Astor et al., 2001; Cerezo, 2009; Dinkes et al., 2009), to be at the first cycle of secondary studies (Serrano and Iborra, 2005); males as more likely to become victims, shyness, little self-control, low self-esteem, high anxiety, etc. (Cerezo, 2009); provocative victim involved in other risky behaviors such as drugs/consumption (Kaltiala-Heino et al., 2000; Cerezo and Méndez, 2009; Tharp-Taylor et al., 2009).

As family protective factors against victimization, some studies underline that adolescents may perceive an over protective family environment, organization and control. Such an over protection would imply a great difficulty to face arrogant or abuse attitudes (Ortega, 2000; Samper-García et al., 2015). Studies have shown that sibling relationships are considered a source of risk or of protection against violence or victimization depending on the sibling size (Piñero-Ruiz et al., 2012).

Traditionally, literature on bullying points out that it is school adaptation what predicts the role of victim. Some studies have evidenced that failure to adapt to school promotes aggressive behaviors as opposed to victimization (Cerezo, 2009; Méndez and Cerezo, in press). As far as the child’s interaction with the peer group diminishes, the child may become more and more isolated and socially rejected (Armas, 2007; Cerezo, 2009; Cerezo and Ato, 2010). Even peer acceptance is recognized as a protection factor against peer victimization (Demaray and Malecki, 2003; Schmidt and Bagwell, 2007).

Thus, the main objective of this study is to analyze the relationship between peer victimization and some risk and protection factors and to identify the differences by role in victimization and compare them with the ones of non-involved bystanders. Some risk and protection factors (personal and environmental) as well as the level of comprehensive maladjustment (personal, at school, in society and with family), often favor or prevent other risk behaviors (consumption of legal drugs and challenging behavior) that shape the victim profile involved in bullying. To this purpose, this research identifies victims’ potential risk and protective factors and compares them with those of non-involved bystanders.

## Materials and Methods

### Participants

Participants in this study were 1.264 students (50.8% female). Age range: 11–18 years old, *M* = 14.41, *SD* = 1.43 (0.2% 11 years old, 11.3% 12 years old,15.7% 13 years old, 22.3% 14 years old, 27.9% 15 years old, 15.7% 16 years old, 5.9% 17 years old and 0.9% 18 years old) in 13 compulsory secondary education institutions. The participants attended public (66.2%) and private/semi-private (33.8%) secondary schools in different geographical areas of the Region of Murcia (72.8% urban and 27.2% rural areas). 83.5% of them were Spanish and 16.5% were foreigners. Distribution by grade: 45.1% (*n* = 557) at first level and 54.9% (*n* = 679) at second level.

### Design and Procedure

This research work is transversal and descriptive. The selection of the participant schools was determined by their acceptance to take part in the study. The participant students were selected from secondary schools in the Region of Murcia, Spain. After obtaining the corresponding permission, students were approached in their own classrooms at school. Researchers explained the objectives of the study and the instruments that would be used. Participation was voluntary and anonymous. The inclusion criteria used were: students in compulsory secondary education, aged between 11 and 18 years. They were requested to attend the school and sit a test that classified them as victims or non-involved bystanders by their own classmates, according to the test Bull-S (Cerezo, 2012). On the other hand, the exclusion criteria were: non-attendance the day the test was passed out, language problems to fully understand the instruments, to be considered an aggressor or a provocative victim by their peers according to the test Bull-S (description in instrument). After obtaining the sample, the selection of individuals was based on the inclusion criteria mentioned above, as it was necessary to focus on the roles of victim and non-involved bystander.

This study was carried out in accordance with the recommendations of the Oviedo Agreement and it was reviewed and approved by the Ethic Committee for clinic research of the University of Murcia. All participants were requested a written informed consent. Parents also gave written informed consent in accordance with the Declaration of Helsinki.

Two sessions of 50 min were used to complete the tests (20 min the Bull-S Test, 20–25 min the second scale, 15–20 min the FRIDA and 30–40 min the TAMAI).

### Data Analysis

In this paper we used a logistic regression (LR) procedure to relate a dichotomous variable (bullying victim/non-involved bystanders) to a set of categorical and continuous variables, which enabled us to identify potential risks and protective factors. In order to analyze the effect of each variable separately, a simple LR (crude odds ratio) was performed. In addition, with the purpose of identifying the variables related to the victim role, a multiple LR and a forward stepwise LR (Wald) were applied. The Odds Ratio (OR) and the 95% confidence interval were calculated in each case. In these multiple models, we have weighted the fit to the model (Hosmer-Lemeshow Test), the significance of coefficients (Omnibus test) as well as an estimation of the (pseudo) determination coefficient (CoxSnell and Nagelkerke). All analyses were run with SPSS 19.0.

### Instruments

Students were requested to fill in the following instruments:

First of all, the Bull-S test (version 3.3) Assessment Test of Aggressiveness was used (Cerezo, 2012). It consisted of 15 direct choice Likert items and was addressed to all individuals in the group-class. The test had three dimensions:

– Dimension 1: Sociometric status (four items by peer nominations). It included a nominal variable that measured individual social status in the group (leader, popular, very rejected, rejected, isolated, controversial, and average). It also included two quantitative variables: the social impact (ISI) indicates the percentage of peers related to each student and the social preference (SPS), which represents the difference between the peers who have assessed a student positively and those who have done so negatively. It also provides information on the level of cohesion in the group-class.
– Dimension 2: Bullying dynamic (six items by peer nominations). It provided information on the students who stood out in at least 25% of each profile linked to bullying dynamics. The features associated to the aggressor profile were related to continuous items: physical strength, aggressiveness and provoking behavior; and those associated with the victim role: cowardice, victimization and fixation. Individuals who scored significantly high in victimization and fixation were classified as provocative victim. In addition, we obtained a qualitative variable reporting on the role assumed in bullying: aggressor, victim, provocative victim or non-involved students (non-involved bystanders).
– Dimension 3: Situational perception (5 Likert scale items). It analyzed the situational aspects in aggressive relationships among peers: type of aggression (insults and threats, physical abuse, rejection and others), place of the aggression (classroom, playground, corridors, and others), frequency of the attacks (never, once or twice a week, rarely or everyday), seriousness of the attacks (not serious at all, hardly serious, serious, quite serious or very much serious), security at school (not safe at all, hardly safe, average, quite safe or very much safe).

The test included socio-demographic variables too. Gender (male/female), age, grade, origin (Spanish/foreigner), course repetition (yes/no), nature of the school (public/private/semi-private) and geographical location (urban/rural) were also collected as variables. Cronbach’s alpha coefficient was 0.68 for total scale scores (73 for aggressors and 0.84 for victims) (Cerezo, 2012). In this study, the coefficient was 0.68 for total scale scores (0.83 for aggressors and 0.84 for victims). Example of items: Whom would you choose as a classmate in the classroom?

The second scale we applied (Méndez et al., unpublished) was based on the “National Survey on Drug Consumption in Secondary School Students” (ESTUDES), issued by the Government Delegation for the National Drug Plan –Delegación del Gobierno para el Plan Nacional sobre Drogas [DGPNSD] (2008) to detect substance use among adolescents in educative contexts. It included 19 dichotomous items about drug consumption and other behaviors. The scale consisted of two factors. Factor I – “Substance Abuse and Health Consequences” – was based on the use of illegal drugs; a higher score indicated a greater possibility of health risk behaviors (have you either participated in any fighting or suffered or initiated any physical attack?, have you been arrested by the police, expelled from school for one or more full days or carry out activities that put your health at risk?) and illegal drug consumption. And Factor II – “Legal Drug Consumption and Challenging Behavior” –, where a higher score indicated a greater possibility of challenging behaviors (Have you had a major conflict or argument with parents or siblings? have you run away from home for more than a day?) and legal drug consumption. Cronbach’s alpha reliability coefficient for total scale scores was 0.64 (0.63 for Factor I and 0.64 for Factor II). The Bartlett statistics were good indicators that a matrix of tetrachoric correlations could be subject to EFA Bartlett (190) = 4269.1, *p* < 0.001, and KMO index, KMO = 0.82. Each factor consisted of different items with a factorial loading > 0.30. The total variance explained by two factors was 58.3%. Example of items: Have you ever smoked a cigarette? Yes/No.

In the third place, we used FRIDA – Interpersonal Risk Factors for Drug Consumption in Adolescence (Secades et al., 2006). It consisted of 90 items in a Likert scale (3 or 5 points), providing a global index of vulnerability or risk and measuring seven factors. Factor 1 (α = 0.88) – “Family Reaction against Drug Consumption” – higher values indicate lower family reaction; for example, the family does not get annoyed if the child is discovered to be smoking. Factor 2 (α = 0.86) – “Peers” – it evaluates friends’ attitude toward drug consumption, friends’ drug consumption and risk activities; higher levels indicate friends have a higher permissive attitude toward drugs and may even be drug consumers. Factor 3 (α = 0.89) – “Access to drugs” – it evaluates how easily adolescents access drugs; the higher the value, the easier the access; Factor 4 (α = 0.64) – “Family Risks” – it inquiries into family relationships, drug consumption and family conflicts; higher values indicate more family conflicts and drug consumption. Factor 5 (α = 0.85) – “Family Education about Drugs” – evaluates the amount of information adolescents receive from their families about drugs; high values indicate a lack of rules about drug consumption. Factor 6 (α = 0.74) – “Family Protective Activities” – includes leisure and sport activities and measures the quality of relationships and academic achievement; higher values indicate less protective activities. Factor 7 (α = 0.70) – “Parental Educational Styles” – it reports on how authoritarian or permissive the parenting style is (higher scores indicate more permissiveness, while lower scores indicate a democratic style and moderate scores an authoritative one). Cronbach’s alpha reliability coefficient was 0.925 for total scale scores (Secades et al., 2006). In this study, the reliability coefficient was 0.81 and in each dimension: Factor 1 (α = 0.88), Factor 2 (α = 0.80), Factor 3 (α = 0.90), Factor 4 (α = 0.79), Factor 5 (α = 0.71), Factor 6 (α = 0.85), and Factor 7 (α = 0.83). Example of item: My best friend smokes. A Not at all, B Occasionally, C Sometimes, D Often.

The fourth scale we used was the Multifactorial Self-evaluation Child Adaptation Test -TAMAI- (Hernández- Guanir, 2015); it consists of 175 dichotomous items that measure five factors. Factor P (α = 0.85) – “Personal Maladjustment,” a high score reports a lack of self-acceptance. Factor E (α = 0.86) – “School Maladjustment,” a high score indicates a lack of satisfaction at school, the appearance of disruptive behavior in the classroom and negative attitudes toward learning. Factor S (α = 0.75) – “Social Maladjustment,” a high score means poor social abilities showing apprehension or distrust. Factor F (α = 0.75) – “Family Maladjustment” – a high score implies a lack of satisfaction with home environment and parents relationship. Factor IH (α = 0.70) – “Sibling Maladjustment” – a high score indicates a lack of satisfaction with sibling interaction. Cronbach’s alpha reliability coefficient was 0.92 for the total scale scores (Hernández- Guanir, 2015). In this study, the reliability coefficient was 0.80 and in each dimension: Factor P (α = 0.71), Factor E (α = 0.83), Factor S (α = 0.70), Factor F (α = 0.70) Factor IH (α = 0.64). Example of item: I have few friends (A) YES (B) NO.

## Results

The distribution of roles in bullying issues among the 1,264 adolescents we studied was as follows: 125 (9.9%) victims, 109 (8.6%) aggressors 7 (0.6%) provocative victim and 1,023 (80.9%) non-involved bystanders. In order to identify possible risk and protection factors in the victim role, a LR analysis was conducted. We compared 125 young victims with 1023 non-involved bystanders. **Table 1** shows the categorical and quantitative variables used for the LR procedure. Firstly, a simple LR analysis (crude) enabled us to detect the individual effect of each variable in the role of victim. The variables in the table proved to be significant in the simple LR (crude). The following risk factors resulted statistically significant: (1) Perceived attitude in friends toward access to drugs (OR = 1.879): students who perceive that their friends would have a moderate easy access to drugs are more likely to be victims than those who perceive little facility; (2) a difficult access to drugs (OR = 2.667): students with easy access to drugs are more likely to fit the profile than those who perceive it as difficult; (3) Parenting style (OR: 2.995): an authoritarian education style can result in three times more risk than a democratic style; (4) Compulsory Secondary Education level (OR = 1.531): undergraduate students (youth) are more likely to become victims than students in the second cycle; (5) Sex (OR = 4.066): being male multiplied the risk by 4 when compared to females; (6) Student social status (OR = 8.280): students ‘rejected’ by their peers have eight times more risk of becoming a victim than other students; (7) Social maladjustment (OR = 1.062): students with higher social maladjustment show a higher risk of becoming victims; (8) Challenging behaviors (OR = 0.848): lower challenging behavior increases the possibilities of becoming a victim; (9) School maladjustment (OR = 0.968): students with higher school adjustment are more likely to become victims. On the other hand, there were significant protection factors: (1) Being popular among peers (OR = 0.218), compared to average students; (2) Age (OR = 0.848): the older peers take a lower risk; and (3) Social Preference (OR = 0.872): those elected by fellow students show a lower risk.

**Table 1:** Logistic regression for victim role.

Categorical variable			Logistic regression: B/OR (95% CI)			
	Category	*N*	%	Crude	Adjusted	Forward selection
Peers	Low	33	9.1	Ref^∗∗∗^	Ref	Ref^∗∗^
	Moderate	512	15.8	0.631/1.879 (0.560–6.304)	0.272/1.313 (0.242–7.114)	1.104/3.018 (0.756–12.046)
	High	603	6.8	-0.315/0.615 (0.214–2.492)	-0.243/0.785 (0.143–4.293)	0.381/1.523 (0.371–6.264)
Access to drugs	Low	175	7.4	Ref^∗∗∗^	Ref	
	Moderate	643	8.4	0.133 / 1.142 (0.608–2.145)	0.386/1.471 (0.692–3127)	
	High	329	17.6	0.981/2.667^∗∗^ (1.417–5.018)	0.754/2.126 (0.986–4.584)	
Parental educational styles	Democratic	50	6.0	Ref^∗∗^	Ref	
	Authoritative	334	15.9	1.083/2.995 (0.887–9.845)	0.906/2.473 (0.606–10.096)	
	Permissive	764	9.0	0.442/1.555 (0.472–5.128)	0.804/2.235 (0.554–9.021)	
Grade	Second	621	9.2	Ref^∗^	Ref	
	First	500	13.4	0.426/1.531^∗^ (1.053–2.227)	-0.103/0.902 (0.463–1.758)	
Gender	Female	617	5.0	Ref^∗∗∗^	Ref^∗∗∗^	Ref^∗∗∗^
	Male	531	17.7	1.403/4.066^∗∗∗^ (2.660–6.216)	1.380/3.974^∗∗∗^ (2.449–6.427)	1.416/4.157^∗∗∗^ (2.573–6.715)
Status	Average	778	9.3	Ref^∗∗∗^	Ref	
	Leader	24	0	-18.920/0	-17.210/0	
	Popular	92	2.2	-1.524/0.218^∗^(0.053–0.903)	-0.308/0.735 (0.180–4.259)	
	Rejected	83	45.8	2.114/8.280^∗∗∗^ (5.046–13.587)	-0.342/0.710 (0.292–1.727)	
	Isolated	171	7.6	-0.215/0.807(0.436–1.493)	-0.086/0.917 (0.476–1.767)	
**Continous Variable**	**Victim(125)**	**Non-involved (1023)**				

Age	14.10 (1.45)^a^	14.44 (1.43)^a^		-0.165/0.848^∗^ (0.744–0.966)	-0.132/0.876 (0.697–1.101)	
Legal drug consumption and challenging behavior (Factor II)	1.74 (1.64)	2.36 (2.12)		-0.163/0.850^∗∗^ (0.767–0.942)	0.010/1.010 (0.884–1.154)	
School maladjustment	9.86 (6.03)	11.31 (6.87)		-0.032/0.968^∗^ (0.941–0.996)	-0.052/0.949^∗^ (0.910–0.990)	-0.061/0.943^∗∗^ (0.905–0.982)
Social maladjustment	9.74 (5.09)	8.23 (4.90)		0.060/1.062^∗∗∗^ (1.023–1.101)	0.093/1.103^∗∗∗^ (1.050–1.159)	0.099/1.105^∗∗∗^ (1.053–1.160)
Social preference (SPS)	-7.14 (11.62)	2.47 (6.32)		-0.137/0.872^∗∗∗^ (0.850–0.895)	-0.132/0.877^∗∗∗^ (0.839–0.916)	-0.121/0.885^∗∗∗^ (0.862–0.910)
				
					HLT = 8.793; *p* = 0.360 OT = 212.262; *p* < 0.001 R^2^Cox-Snell = 0.173 R^2^Nagelkerke = 0.344	HLT = 6.566; *p* = 0.584 OT = 208.842; *p* < 0.001 R^2^Cox-Snell = 0.164 R^2^Nagelkerke = 0.327

*N: Total cases. %: Victim percent. B/OR (95% CI): Regression coefficient/Odds Ratio (95% confidence interval). ^a^Mean (standard deviation).*

*^∗^*p* ≤ 0.05, ^∗∗^*p* ≤ 0.01, ^∗∗∗^*p* ≤ 0.001. HLT, Hosmer Lemeshow Test. OT, Omnibus test. Ref, Reference value. Ref^∗^, Significance level of categorical variable.*

Subsequently, a multiple LR analysis (Adjusted) was accomplished, aiming to identify risk/no protection or non-redundant factors. With this procedure, the following simultaneous risk factors to become a victim were identified: being male, school adapted, socially maladjusted and slightly preferred by their peers.

Last, aiming at the exclusion of irrelevant or redundant factors, some variables were selected with a Forward LR (Wald statistics) procedure (Forward selection), which confirmed the aforementioned factors, including an additional one: Perception of the friends’ attitude toward access to drugs. Students who perceive that their friends would have a moderate or easy access to drugs are more likely to become victims.

These results are similar if analyzed separately for boys and girls.

## Discussion

Understanding the factors that predict peer victimization at school requires a close examination of the complex inter-relationships between the individual and his/her environment. In this study, a number of factors related to victimization in secondary education adolescents have been identified.

Concerning social environment, the results of our study show that adolescents who have less drug-friendly friends are more likely to be potential victims than those who show a higher tolerance. The results obtained in relation to social environment are consistent with other research works that show adolescents can be influenced by their group of friends on drugs consumption (Sánchez et al., 2007; Alfonso et al., 2009; Pérez-Fuentes and Gázquez, 2010; Delegación del Gobierno para el Plan Nacional sobre Drogas [DGPNSD], 2014), especially through best friend (Espada et al., 2008), and even promote the perception of easy access to its consumption (Cerezo et al., 2013).

In this sense, our findings point out that those adolescents who perceive easier access to drugs might be at greater risk of becoming victims. Nevertheless, the victim profile is not usually involved in challenging behaviors (i.e., legal drug consumption, have you had a major conflict or argument with parents or siblings, run away from home for more than a day?), unlike studies on the aggressor profile, provocative victim or non-involved bystanders (Kaltiala-Heino et al., 2000; Cerezo and Méndez, 2013). Probably, this difference between group values and individual’s behavior makes perception become a risk factor to become a victim.

In relation to family environment, the results obtained regarding the parenting style support revealed that children exposed to peer victimization have a different home environment than those who are not. Children whose parents show an authoritarian style run a greater risk to become a victim than those coming from permissive and democratic family environments. In contrast, some studies showed that permissive parental style predicts the experience of victimization while the authoritarian parental style best predicts bullying behavior (Baldry and Farrington, 2000; Georgiou and Stavrinides, 2013). Therefore, it will promote victimization and inhibit the attachment to peers (Ortega, 2000; Samper-García et al., 2015).

Regarding personal features, our data show that students at the first cycle of secondary school are more likely to be at risk than those at the second, which also confirms the decreasing risk associated to the variable age. Older students are more likely to experience bullying than younger school students and perceive school as unsafe as a number of studies have shown (Astor et al., 2001; Cerezo, 2009; Dinkes et al., 2009). Serrano and Iborra (2005) consider the probability to become a victim is more likely to happen during the 1st years of secondary studies while it tends to decrease in the following years. These studies are coherent with our data that point out age as a protective factor. The protection and social skills that adolescents develop with age explained data presented in this study.

Researchers are careful about conclusions on gender differences in bullying behavior (Hong and Espelage, 2012). Previous findings indicated that boys are usually either victims or authors of direct forms of bullying while girls experience indirect bullying (Olweus, 1993; Varjas et al., 2009). Cerezo (2009) points out males as more likely to become victims. Our study indicates that males are four times more likely than females to become victims.

Regarding student social status, other studies prove that non-involved children are better placed in their social networks than those involved in bullying dynamics (García-Bacete et al., 2010). According to our results, adolescents rejected by their peers are at higher risk, up to 45 times higher, than the average student is. Among involved children, aggressors get more support and are more accepted by their peers than victims (Estévez et al., 2007; Salmivalli, 2010; Van der Schoot et al., 2010). Victims are rejected, when not ignored, by most of the group members (Cerezo and Ato, 2010) which certainly contributes to their helplessness (Ortega, 2000). This finding supports the results obtained in this study.

Cerezo and Ato (2010) point out that victim were worse placed than aggressors in the network of interpersonal relationships. That is, both victims and aggressors are rejected but victims are also considered cowards. Regarding social perception, victims reported to be lonely, nobody caring about them, because the rest is not concerned about the seriousness of the situation, and that could encourage the persistence of bullying. In this line, this research consistently shows that students rejected by their peers run 8 times more risk of becoming a victim than average students.

The data we have obtained show that social maladjustment increases the risk of victimization while school maladjustment reduces it (therefore, adolescents with a higher level of school adjustment are also at higher risk). High achievement is usually linked to school adjustment. Students with the highest achievement are more rejected than average students. This may help to interpret our data. In addition, at school environments, the victim role shows a higher academic achievement than the aggressor role, being similar to the average achievement of the peer group/classroom (non-involved bystanders) (Cerezo, 2009; Méndez and Cerezo, in press).

The last related variable is social preference. The risk adolescents run diminishes as they are more socially accepted. Preference by peers, popularity, and friendship are very important for adolescents (Espelage, 2002). Besides our findings, other studies found friendship to be a protection against victimization (Demaray and Malecki, 2003; Schmidt and Bagwell, 2007).

Both adjusted LR and forward selection procedures identify the same subgroup of significant variables in relation to the victimization and can define the test type features in the sample under study: to be male, to perceive that friends are not at high risk of drug consumption, low school maladjustment, high social maladjustment and low social acceptance by peers. The identified variables, however, explain only 34%, at the most, of the variation on victimization. This moderated percentage underlines the complexity of the issue. Other studies point to different risk factors that were not studied but should be considered in future studies. Some personal factors are shyness, little self-control, low self-esteem, high anxiety, depression, race or ethnicity, handicaps, learning disabilities (Cerezo, 2009), challenging victim involvement in other risky behaviors (Kaltiala-Heino et al., 2000; Cerezo and Méndez, 2009; Tharp-Taylor et al., 2009); some contextual factors as: (a) over protective family environment (Ortega, 2000), hierarchical relationships among siblings (Piñero-Ruiz et al., 2012); negative peer relationships (Salmivalli, 2010); (b) school environment features, such as the lack of resources or little experienced teachers (Serrano and Iborra, 2005), inter-parental violence (Corvo and deLara, 2010); and (c) social environment, for instance exposure to violence in the media (David-Ferdon and Hertz, 2007).

These results should have consequences for educational policy and practice. It is necessary to promote inclusion (Llorent et al., 2016). It is also necessary to strengthen emotional education and acquisition of social skills (Sastre, 2016). At school level, it is recommended to provide teachers with resources (Serrano and Iborra, 2005). It is important that society as a whole breaks the law of silence or helplessness (Ortega, 2000), giving an active role to non-involved bystanders. Kärnä et al. (2010) suggest that non-involved behaviors in bullying situations moderate the effects of individual and interpersonal risk factors for victimization. Influence on these behaviors might be an effective way to protect vulnerable children from victimization.

It is convenient to take into account teachers and family’s perspectives and even gather information on other behaviors that may be influencing victimization, such as personality, self-esteem, or self-concept, and to collaborate with specialists when dealing with medical problems or psychological consequences of victimization.

There have been several meta-analyses and studies on bullying prevention and intervention programs. Results indicated moderate effect sizes on self-reported victimization that students experienced from aggressors (Smith et al., 2004). Hong and Espelage’s (2012, p. 2012) social-ecological approach considered that *responses to aggressors, rather than rely on traditional punitive measures, should approach both aggressors and victims patterns of behavior, with particular attention to non-involved bystanders at school, as well as the classroom-social climate and other influences such as family, community and society. Maybe, intervention programs have been too focused on aggressors and rarely on victims and non-involved bystanders.*

Researchers also noted that anti-bullying programs were more efficient when implemented with older students (i.e., 11 and older) (Smith et al., 2004). In spite of the large number of prevention programs implemented in our country: “Educating in harmonious coexistence in order to prevent violence” (Ortega, 2000); “Aid between peers Program” (Cowie and Fernández, 2006); KiVa antibullying program (Kärnä et al., 2011); “System to detect racial-based Bullying through Gamification” (Álvarez-Bermejo et al., 2016), “Using a 3D simulation Instrument in educational settings” (Cangas et al., 2016). We must insist in prevention programs based on ecological approach that take into account risk and protection factors. Furthermore, intervention programs should address victims and non-involved bystanders instead of only aggressors. In addition, it should include risky behaviors related to bullying dynamics, like the consumption of drugs.

Like many of the existing studies on the topic of bullying and peer victimization, the present study used a standard cross-sectional methodology. Even though this is an established method in social sciences, it also shows limitations, such as significant constraints in unfolding cause and effect relationships. Our conclusions are limited because they are based on correlational relationships. Additional research on these variables with longitudinal data is needed.

## Author Contributions

IM: Fieldwork, theoretical development and writing. CR: Theoretical development and writing. JL: Fieldwork and methodological treatment.

## Conflict of Interest Statement

The authors declare that the research was conducted in the absence of any commercial or financial relationships that could be construed as a potential conflict of interest.
